# 2‐dodecyl‐6‐methoxycyclohexa‐2,5‐diene‐1,4‐dione mediates the effect of ROS‐enhanced PI3K/Akt/mTOR pathway on autophagy in breast cancer

**DOI:** 10.1002/2211-5463.13940

**Published:** 2024-12-09

**Authors:** Linqian Chen, Meifeng Chen, Yan Xie, Yuyan Zhang, Shutian Mo, Yongfei He, Tianyi Liang, Yuan Liao, Renbin Huang, Guodong Huang, Chuangye Han, Thi Thai Hoa Pham

**Affiliations:** ^1^ Zhuang & Yao Medicine Research and Development Center Guangxi International Zhuang Medicine Hospital Affiliated to Guangxi University of Chinese Medicine Nanning China; ^2^ Guangxi Medical University School of Pharmacy Nanning China; ^3^ Department of Hepatobiliary Surgery The First Affiliated Hospital of Guangxi Medical University Nanning China; ^4^ Department of Hepatobiliary and Pancreatic Surgery The Eighth Affiliated Hospital of Sun Yat‐sen University Shenzhen China; ^5^ The Second Affiliated Hospital of Guangxi Medical University Nanning China; ^6^ Guilin Medical College School of Pharmacy Guilin China; ^7^ Guangxi Key Laboratory of Early Prevention and Treatment for Regional High Frequency Tumor Nanning China; ^8^ Guangxi Key Laboratory of Enhanced Recovery After Surgery for Gastrointestinal Cancer Nanning China

**Keywords:** autophagy, breast cancer, 2‐dodecyl‐6‐methoxycyclohexa‐2,5‐diene‐1,4‐dione, PI3K/Akt/mTOR signaling pathway, ROS

## Abstract

Several studies have suggested a potential antitumor effect of 2‐dodecyl‐6‐methoxycyclohexa‐2,5‐diene‐1,4‐dione (DMDD). To further understand the mechanism of action of this compound, we investigated its effect on the phosphatidylinositol‐3‐kinase (PI3K)/serine–threonine kinase (Akt)/mammalian target of rapamycin (mTOR) signaling pathway. We show that DMDD application significantly inhibited the proliferation of breast cancer cell lines MDA‐MB‐231 and ER‐α positive MCF‐7. Furthermore, DMDD application resulted in increased intracellular reactive oxygen species (ROS) levels, apoptosis and autophagy, whereas it downregulated the expression of PI3K, Akt and mTOR mRNA and proteins, and increased the expression of LC3II/I and p62 proteins. In a mouse breast cancer xenograft model, DMDD inhibited tumor growth. Expression analyses suggest that ROS levels were higher in DMDD treated tumor tissues, whereas immunohistochemical analyses suggest that apoptotic cells were more prevalent in the DMDD treated group compared to the control group. Taken together, our results suggest that the molecular mechanism of action of DMDD may involve the enhancement of breast cancer autophagy through the PI3K/Akt/mTOR signaling pathway by mediating ROS expression.

## Graphical abstract

2‐dodecyl‐6‐methoxycyclohexa‐2,5‐diene‐1,4‐dione (DMDD) is isolated from the roots of *Averrhoa carambola* L. and exhibits antioxidant, anti‐hyperglycemic, anti‐hyperlipidemic and anti‐tumor properties. This study suggests that DMDD inhibits the growth of breast cancer through the phosphatidylinositol‐3‐kinase (PI3K)/serine–threonine kinase (Akt)/mammalian target of rapamycin (mTOR) signaling pathway *in vitro* and *in vivo*. Graphic abstract typesetting design inspiration (https://doi.org/10.1002/2211‐5463.13914).

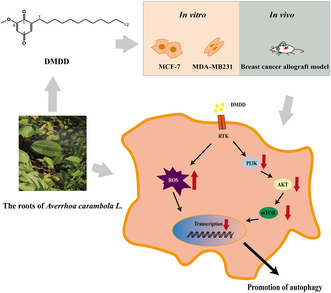

**Abbreviations**
Akt: serine–threonine kinase
ANOVA: analysis of variance
CCK: cell counting kit
DMDD: dodecyl‐6‐methoxycyclohexa‐2,5‐diene‐1,4‐dione
DMEM: Dulbecco's modified Eagle's medium
HE: hematoxylin and eosin
mTOR: mammalian target of rapamycin
MTT: methyl thiazolyl tetrazolium
PBS: phosphate‐buffered saline
PI3K: phosphatidylinositol‐3‐kinase
PTX: paclitaxel
qRT‐PCR: quantitative real‐time
ROS: reactive oxygen species
TUNEL: terminal deoxynucleotidyl transferase dUTP nick end labeling

According to the American Cancer Society and cancer incidence and mortality data from the International Agency for Research on Cancer in GLOBOCAN 2022, breast cancer has the highest incidence rate among women, making it the most prevalent and widespread malignancy globally [1, 2]. Breast cancer exhibits significant metastatic and invasive properties [3], particularly in triple‐negative and HER‐2 negative breast cancer cells [4, 5]. Conventional therapies, including surgery, radiotherapy and chemotherapy, have demonstrated efficacy in certain patient populations; however, their inherent limitations cannot be overlooked [6]. Therefore, the development of potential medicinal agents to combat tumor metastasis remains a key objective in breast cancer research.

The initiation and regulation of cancer involve two primary pathways: apoptosis and autophagy [7]. Autophagy can both promote and inhibit tumor function and is intricately linked to apoptosis and programmed cell death. The signaling pathways of these two processes are interconnected through various crosstalk mechanisms. Recent insights suggest utilizing autophagy mechanisms and related small‐molecule regulators to develop potential anti‐tumor agents that enhance therapeutic effects [8]. The anti‐tumor effects of natural substances have become a prominent focus in both domestic and international research. Natural chemical compounds can activate pro‐autophagy and pro‐apoptotic pathways [9]. For example, magnolol, an active component derived from Magnolia bark, induces apoptosis and cell cycle arrest in U87 MG glioma cells [10]. The Chinese herbal medicines *Magnolia* and *Aristolochia* contain quaternary alkaloids, collectively referred to as magnolia. This compound can increase the susceptibility of breast cancer cells to doxorubicin and enhance its anti‐cancer effects by inducing apoptosis and autophagy in these cells [11].

2‐dodecyl‐6‐methoxycyclohexa‐2,5‐diene‐1,4‐dione (DMDD) is isolated from the roots of *Averrhoa carambola L*. and exhibits antioxidant, anti‐hyperglycemic, anti‐hyperlipidemic and anti‐tumor properties [12, 13]. Research indicates that DMDD (Fig. 1) can inhibit the growth of breast tumors in mice and the proliferation of cancer cells, including those associated with lung cancer, breast cancer and cholangiocarcinoma [14, 15, 16]. This study investigates the effects of DMDD on triple‐negative cells (MDA‐MB‐231), HER2‐negative cells (MCF‐7) and mouse tumors. DMDD is a novel autophagy inducer that enhances the phosphatidylinositol‐3‐kinase (PI3K)/serine–threonine kinase (Akt)/mammalian target of rapamycin (mTOR) signaling pathway by modulating reactive oxygen species (ROS).

**Fig. 1:**
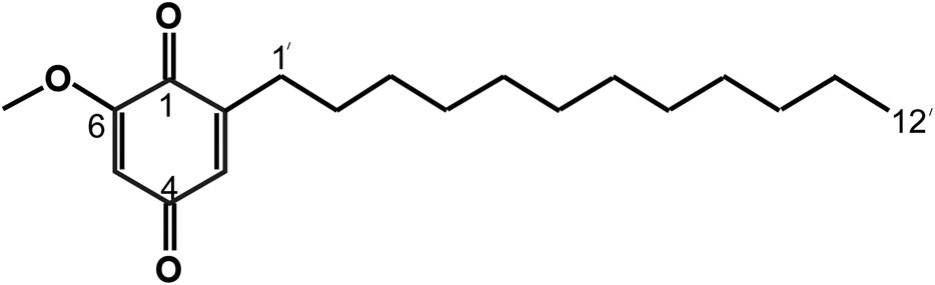
2‐dpdecyl‐6‐methoxycyclohexa‐2,5‐diene‐1,4‐dione (DMDD) chemical constitution.

## Materials and methods

### Cell culture

The breast cancer cell lines MDA‐MB‐231 (triple‐negative, cell catalog number: TCHu227), MCF‐7 (HER2‐negative, cell catalog number: TCHu 74) and 4T1 (cell catalog number: TCM32) were obtained from the Cell Bank of the Shanghai Academy of Life Sciences (Shanghai, China). All cells were maintained in Dulbecco's modified Eagle's medium (DMEM) (KGM12800N‐500; KeyGen BioTech, Inc., Nanjing, China), supplemented with 10% fetal bovine serum (FBS‐CE500; NEWZERUM, Inc., Staffordshire, Christchurch, New Zealand), 100 μg·mL^−1^ streptomycin and 100 U·mL^−1^ penicillin G (P1400; Solarbio Biotechnology, Inc., Beijing, China). Cells were incubated in a humidified atmosphere of 5% CO₂ at 37 °C, and were subcultured every 3 days, with a change to fresh media every 24 h.

### Drugs and antibodies

DMDD was prepared by our research group [17]. Paclitaxel (PTX) was acquired from Aladdin Inc. (P106868‐10 mg; CAS:33069‐62‐4; Shanghai, China). PI3K (dilution 1 : 800; catalog. no. 20584‐1‐AP; polyclonal antibody; origin of the antibody: Proteintech Group, Inc., Wuhan, Hubei, China), Akt (dilution 1 : 25000; catalog. no. 60203‐2‐Ig; monoclonal antibody; origin of the antibody: Proteintech Group, Inc.), mTOR (dilution 1 : 25 000; catalog. no. 66888‐1‐Ig; monoclonal antibody; origin of the antibody: Proteintech Group, Inc.), p62 (dilution 1 : 5000; catalog. no. 18420‐1‐AP; polyclonal antibody; origin of the antibody: Proteintech Group, Inc.), LC3 (dilution 1 : 500; catalog. no. 18725‐1‐AP; polyclonal antibody; origin of the antibody: Proteintech Group, Inc.), ACTIN (dilution 1 : 50 000; catalog. no. 66009‐1‐Ig; monoclonal Antibody; origin of the antibody: Proteintech Group, Inc.). Horseradish peroxidase‐conjugated secondary antibodies (dilution 1 : 1000; A0516; origin of the antibody: Beyotime Biotechnology, Inc., Shanghai, China) were used.

### Methyl thiazolyl tetrazolium (MTT) cell viability assay

The MTT assay was employed to assess the viability of human breast cancer MDA‐MB231 and MCF‐7 cells. The MTT colorimetric method (M1020; Solarbio Biotechnology, Inc.) was employed to evaluate the proliferation of MDA‐MB231 and MCF‐7 cells. Cells of human breast cancer MDA‐MB231 (7.0 × 10^4^ cells·mL^−1^) and MCF‐7 (8.0 × 10^4^ cells·mL^−1^) in the logarithmic growth phase were counted and then seeded into 96‐well plates at a volume of 100 μL per well. The cells were incubated overnight in a CO₂ incubator at 37 °C. The control and experimental groups, treated with various concentrations of DMDD (1, 5, 10, 15, 20, 40 and 80 μmol·L^−1^) and PTX (0.1, 0.5 and 1 μmol·L^−1^), were incubated for 24 and 48 h. The absorbance of each well was measured using a microplate reader (Synergy H1; Agilent, Santa Clara, CA, USA) at a wavelength of 490 nm. The half maximal inhibitory concentration (IC₅₀) of DMDD on MDA‐MB231 and MCF‐7 cells was calculated. The optimal concentration of PTX was selected for use in the positive control group. All experiments were performed in triplicate.

The experimental groups and administration concentrations were:

1. Control group (CK): 0 μmol·L^−1^ (μm);
2. Paclitaxel group (PTX): 1 μm;
3. Low dose group of DMDD (DMDD‐L): 2.5 μm (MDA‐MB231), 6.25 μm (MCF‐7);
4. Medium dose group of DMDD (DMDD‐M): 5 μm (MDA‐MB231), 12.5 μm (MCF‐7);
5. High dose group of DMDD (DMDD‐H): 10 μm (MDA‐MB231), 25 μm (MCF‐7).

### Colony formation assay

The colony formation assay was employed to evaluate the population dependence and proliferative capacity of the cells. MDA‐MB231 and MCF‐7 cells were assigned to control, PTX, DMDD‐L, DMDD‐M and DMDD‐H groups. Each group of cells received the corresponding concentrations of DMDD and was cultured for 24 h. Cells from each group were collected. Approximately 1 mL of medium containing 500 cells per well was added to a six‐well plate and cultured under standard conditions. After 2 weeks, the cells on the culture plate were fixed with anhydrous ethanol for 10 min and stained with 0.1% crystal violet (C0121‐100 mL; Beyotime Biotechnology, Inc.) for 10 min. Colony‐forming units were photographed, and three random views from each well were inspected to calculate the area of cell colonies using imagej, version 1.53t (National Institute of Health, Bethesda, MD, USA) [18]. Each experiment was conducted independently three times. Results are presented as relative quantification compared to the control group cells.

### Cell migration and invasion assays

The ability of cell migration was evaluated. MDA‐MB231 and MCF‐7 cells were treated with varying concentrations of DMDD for 24 h. For these assays, approximately 5.0 × 10^4^ cells in 200 μL of serum‐free DMEM were seeded onto filter inserts (cell culture inserts; BD Biosciences, Franklin Lakes, NJ, USA), with 600 μL of medium containing 10% fetal bovine serum added to the lower chamber. After 24 h of incubation, the upper chamber was washed with phosphate‐buffered saline (PBS) to remove any non‐migrated cells. Cells were fixed on the lower side of the chamber using 4% paraformaldehyde (P0099‐100 mL; Beyotime Biotechnology, Inc.) and stained with 0.1% crystalline violet (C0121‐100 mL; Beyotime Biotechnology, Inc.) for 10 min at room temperature. After washing and drying, the cells were photographed and recorded using an inverted microscope (CKX53; Olympus, Tokyo, Japan). Three random views from each membrane were inspected to calculate the area of migrated cells using imagej. Each experiment was conducted independently three times. The results are expressed as relative quantification compared to the control group cells.

The ability of cell invasion was also evaluated. Transwell chambers were coated with Matrigel (C0372‐1 mL; Beyotime Biotechnology, Inc.) and incubated at 37 °C for 3 h to achieve a gel‐like consistency. MDA‐MB231 and MCF‐7 cells were treated with various concentrations of DMDD for 24 h. Cells were collected and resuspended in serum‐free DMEM. Approximately 200 μL of serum‐free medium containing 5.0 × 10^4^ cells was added to the upper chamber, with 600 μL of medium containing 10% fetal bovine serum added to the lower chamber. After 24 h of incubation, the upper chamber was washed with PBS to remove the cells present in it. Cells were fixed on the lower side of the chamber using 4% paraformaldehyde and stained with 0.1% crystalline violet for 10 min at room temperature. After washing and drying, the cells were photographed and recorded using an inverted microscope (CKX53; Olympus). Three random views from each membrane were inspected to calculate the area of invading cells using imagej software. Each experiment was conducted independently three times. The results are presented as relative quantification compared to the control group cells.

### Flow cytometry for evaluation of cell cycle, apoptosis and ROS assays

MDA‐MB231 and MCF‐7 cells in the logarithmic growth phase were seeded into six‐well plates (2.0 × 10^5^ cells) and cultured for 24 h. Different concentrations of DMDD medium were added to the plates and incubated for 24 h. The cell cycle, apoptosis, and intracellular reactive oxygen species were assessed using Accuri C6 BD flow cytometer [19] (BD Biosciences), following the protocols provided by the cell cycle and apoptosis analysis kits (C1052; Beyotime Biotechnology, Inc.), the annexin V‐FITC apoptosis detection kit (C1062L; Beyotime Biotechnology, Inc.) and the reactive oxygen species detection kit (CA1410; Solarbio Biotechnology, Inc.). Flow cytometric data analysis was performed using flowjo, version 10.8.1 (TreeStar, Inc., Ashland, OR, USA). All experiments were conducted in triplicate. The results of the cell ROS assays are presented as relative quantification compared to the control group cells.

### Cellular autophagy vesicle assay

MDA‐MB231 and MCF‐7 cells in the logarithmic growth phase were seeded into six‐well plates (2.0 × 10^5^ cells) and cultured for 24 h. The cells were incubated with varying concentrations of DMDD medium for 24 h. Monodansyladamantanamine [20] was employed as a fluorescent probe to rapidly detect autophagy using the corresponding staining kit (C3018S; Beyotime Biotechnology, Inc.). Staining was observed and photographed using a fluorescence microscope (CKX53; Olympus). Three random views for each well were inspected to calculate the fluorescence intensity of autophagic vesicle cells using imagej. Each experiment was conducted independently three times.

### Western blot analysis

Cells were obtained from six‐well culture plates, and proteins were extracted from the cell lines using RIPA buffer (P0013B; Beyotime Biotechnology, Inc.), supplemented with a protease inhibitor (P1006; Beyotime Biotechnology, Inc.). Cell lysates were centrifuged at 4 °C for 15 min at 12 000 ***g***, and the supernatants were then isolated. Total protein concentration was quantified using a BCA protein assay kit (P0012; Beyotime Biotechnology, Inc.). Equal amounts of protein were loaded into each well and separated on 6–15% SDS/PAGE gels at 110 V for 45 min. These proteins were then transferred onto a hydrophobic poly(vinylidene difluoride) transfer membrane (IPVH00010; Millipore, Merck KGaA Inc., Shanghai, China) using the Mini‐PROTEAN® Tetra cell system (Bio‐Rad Laboratories, Inc., Hercules, CA, USA). Membranes were blocked using QuickBlock™ Blocking Buffer (P0231; Beyotime Biotechnology, Inc.) for 30 min at room temperature. Following blocking, membranes were incubated with primary antibodies (1 : 500) overnight at 4 °C. Subsequently, the membrane was washed three times in PBS containing 0.1% Tween 20 and then incubated with a secondary antibody at a dilution of 1:1000 for 2 h at room temperature. The membrane was washed three additional times, each for 5 min. Protein bands were visualized using Clarity ECL Western Blotting Substrate (P0018FM; Beyotime Biotechnology, Inc.) and the ChemiDoc Imaging System (HVP ChemStudio815; Analytikjena, Inc., Jena, Germany). All experiments were conducted in triplicate.

### Quantitative real‐time PCR (qRT‐PCR) analysis

Total RNA was extracted using RNAiso Plus (code no. 9108/9109; Takara, Shiga, Japan) in accordance with the manufacturer's instructions. The concentration of total RNA was measured using a microplate reader (Synergy H1). Approximately 500 ng of RNA was reverse transcribed using the First Strand cDNA Synthesis kit (code no. RR047A; Takara) on a high‐throughput gradient gene amplification instrument (BIBBY Prime Elite 102, Staffordshire, UK). Quantitative qRT‐PCR was conducted using TB Green® Premix Ex Taq™ II (code no. RR820A; Takara) on a qRT‐PCR system (qTOWER3G; Analytikjena, Inc.) in accordance with the manufacturer's instructions. The PCR cycling conditions were set as: initial denaturation at 94–95 °C for 1–3 min; 25–35 cycles of denaturation at 94–95 °C for 15–30 s, annealing at 4–5 °C below the primer's melting temperature (~50–65 °C) for 20–40 s and extension at 72 °C for approximately 1 min per 1 kb of target DNA. This was followed by a final extension at 72 °C for 5–10 min, and the samples were then stored at 4 °C.

For quantification, the 2^−ΔΔCT^ method [21] was utilized, with GAPDH expression set as an internal standard. Melt‐curve analyses confirmed that all qRT‐PCR products were singular DNA duplexes. All experiments were conducted in triplicate. All the mouse‐ or human‐specific primers were designed and synthesized by Sangon Biotech (Shanghai, China). The sequences of primers are obtained from the PrimerBank database [22] with detailed information listed in Table 1.

**Table 1:** Primer sequences used for PCR.

Gene		5′‐ to 3′
GADPH	Forward	TGACATCAAGAAGGTGGTGAAGCAG
	Reverse	GTGTCGCTGTTGAAGTCAGAGGAG
mTOR	Forward	CAACCAGCCAATCATTCGCATTCAG
	Reverse	ATGTCCGTTGCTGCCCATAAGTG
PI3K	Forward	CTGTGCCTTCTGCCTTACGGTTG
	Reverse	GCAATCGTCGTGGCGTCCTTC
Akt	Forward	GCAGGATGTGGACCAACGTGAG
	Reverse	GCAGGCAGCGGATGATGAAGG

### Animal procedures and ethical statements

BALB/c nude female mice (5 weeks old, weighing 20–22 g) were purchased from Guangxi University of Traditional Chinese Medicine (Nanning, China) (No. 430727231100918116). All experiments were approved by the Animal Protection and Use Committee at Guangxi Medical University (No. 202303008). The animal ethical review adhered to the ‘Guidelines for the Treatment of Laboratory Animals’ issued by the Ministry of Science and Technology of the People's Republic of China, as well as the ‘Guidelines for the Ethical Review of Laboratory Animals – Animal Welfare’ issued by the Chinese National Standard GB/T35892‐2018. The animals were housed in cages, allowing for unrestricted movement and free access to food and water. All animals were housed in humidity‐ and temperature‐controlled rooms (25 °C and 50% relative humidity, respectively) and were exposed to a 12 : 12 h dark/light photocycle. None of the experimental mice became severely ill or died prior to the experimental endpoint.

### Establishment of 4T1 breast cancer allograft model and animal administration

4T1 mouse breast cancer cells were cultured in large quantities until they reached the logarithmic growth phase, digested, and subsequently were collected. Approximately 1.0 × 10^7^ 4T1 cells in a 0.1‐mL suspension were subcutaneously injected into the right axilla of mice to establish a model of subcutaneously transplanted breast cancer. Tumor growth was assessed every other day. When the tumor volume reached 100 mm^3^, the mice were randomly divided into five groups: model, PTX (1 μg·g^−1^, q3d, intratumoral injection), DMDD‐L (DMDD, 4.5 μg·g^−1^, q3d, intratumoral injection), DMDD‐M (DMDD, 9 μg·g^−1^, q3d, intratumoral injection) and DMDD‐H (DMDD, 18 μg·g^−1^, q3d, intratumoral injection) groups. Six mice in each group were treated for 20 days, with tumor volume and weight measured every 2 days. Tumor volume was calculated using the formula: (width^2^ × length)/2. The inhibition rate of tumor growth was calculated as: (%) = (mean tumor weight of the vehicle group − mean tumor weight of the treatment group)/mean tumor weight of the vehicle group × 100%. When the tumor volume reached 2000 mm^3^ or at the end of the experiment, the mice were killed using 20% urethane (N159513‐5g, CAS: 589‐41‐3; Aladdin, Inc., Shanghai, China).  

### Detection of ROS in mouse tumor tissue

A 50‐mg sample of tumor tissue was obtained from each mouse, combined according to the experimental groups to represent the total tumor tissue for each group, and then homogenates were prepared following the instructions of the ROS kit (CA1410; Solarbio Biotechnology, Inc.). The absorbance values of each well were measured using a microplate reader (Synergy H1) at an excitation wavelength of 488 nm and an emission wavelength of 525 nm, with three replicate wells established for each group. The absorbance values from the treatment group and control group were used to compare the effects of DMDD administration on ROS levels in mouse tumor cells. The results for ROS levels in mouse tumor tissue are expressed as relative quantification compared to the control group cells.

### Hematoxylin and eosin (HE) staining

A quarter of the mouse tumors were immersed in 4% paraformaldehyde (P0099‐100 mL; Beyotime Biotechnology, Inc.) for 24 h, followed by dehydration, waxing and sectioning. The sections from each group were dewaxed and stained with HE (C0105S; Beyotime Biotechnology, Inc.) to assess the pathological changes in tumor tissue structure.

### Terminal deoxynucleotidyl transferase dUTP nick end labeling (TUNE) assay

Tumor tissue was fixed, dehydrated, embedded in paraffin and sectioned. Apoptosis was detected following the instructions provided by the TUNEL kit (C1098; Beyotime Biotechnology, Inc.). The apoptotic nuclei were stained brown. Three random fields of view were selected under a light microscope (CKX53; Olympus). The proportion of apoptotic cells was analyzed using imagej software. The average optical density value was calculated and expression levels were evaluated.

### Immunohistochemical analysis

The expression of LC3 and Ki67 in tumor tissues was assessed using immunohistochemistry. The sections were dewaxed, and the antigen was retrieved using a 0.01 mol·L^−1^ citric acid solution at pH 6.0, followed by incubation with 3% hydrogen peroxide. The sections were blocked with non‐immune serum and incubated overnight at 4 °C with LC3 (dilution 1 : 250) and Ki67 (dilution 1 : 5000) antibodies. On the following day, the sections were incubated with goat anti‐rabbit secondary antibody (dilution 1 : 1000), stained using the DAB Horseradish Peroxidase Color Development Kit (P0202; Beyotime Biotechnology, Inc.) and counterstained with hematoxylin. The sections were observed and analyzed using an optical microscope (CKX53; Olympus). The positive expression in immunohistochemistry was analyzed using imagej software and the average optical density value was calculated to evaluate the expression levels.

### Statistical analysis

All experiments were performed in triplicate. All data are presented as the mean ± SD. The results of the cell colony formation assay, cell migration assay, cell invasion assay and ROS assay are presented as the relative values of the experimental group compared to the control group. Therefore, when represented as the mean ± SD, the control group is set to 1, making the relative value of the control group across the three repeated experiments also equal to 1, with the SD value being 0. For *in vivo* experiments, data were obtained from three to six different animals. Data were analyzed using one‐way analysis of variance (ANOVA) followed by Bonferroni post‐hoc tests. *P* < 0.05 was considered statistically signiifcant. All analyses were conducted using prism, version 9.50 (GraphPad Software Inc., San Diego, CA, USA).

## Results

### Effect of DMDD on the proliferation of MDA‐MB231 and MCF‐7 cells

**Fig. 2:**
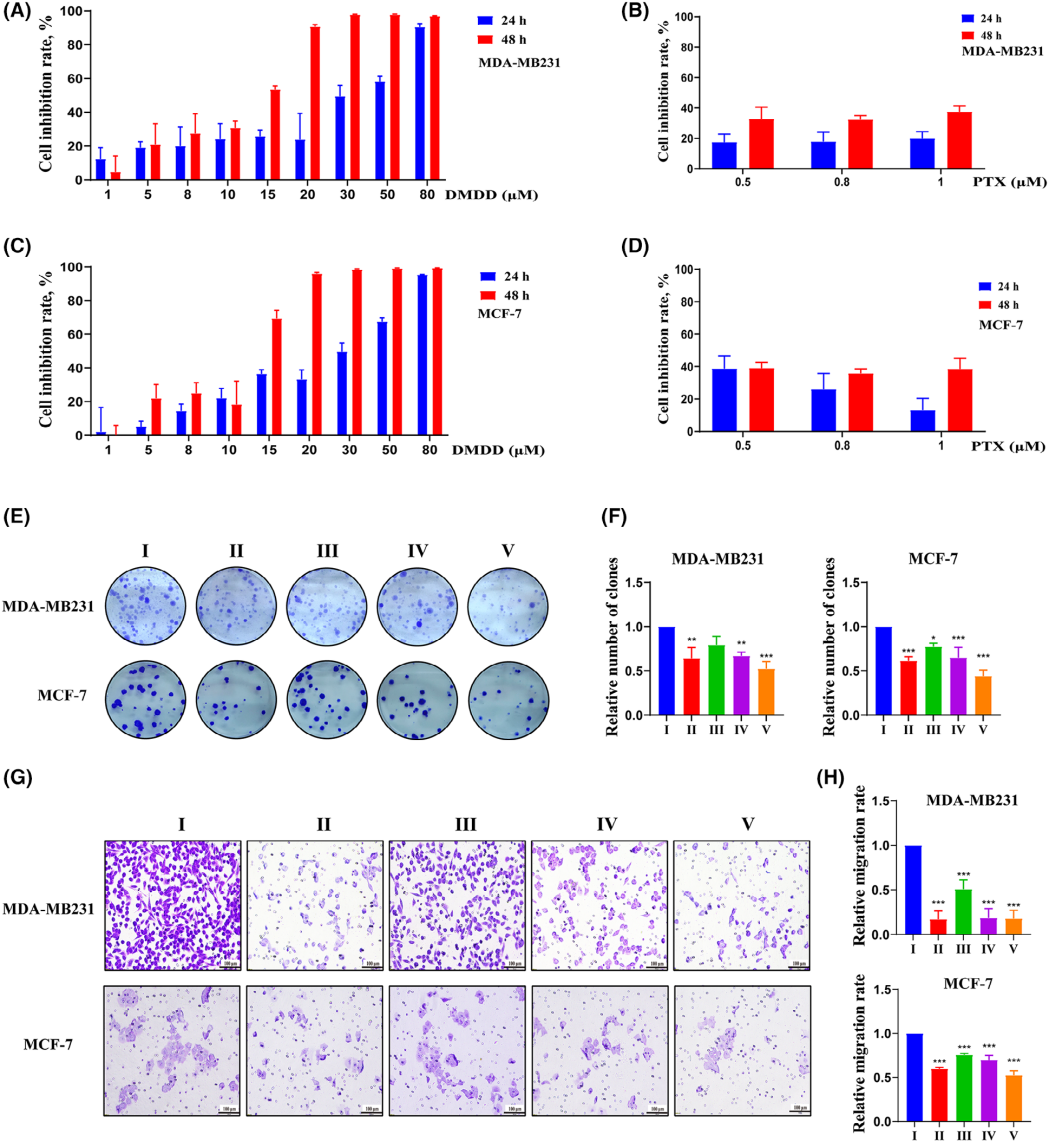
(A, C) Cell viability was analyzed by a cell counting kit (CCK)‐8 assay. The cells were incubated with DMDD at different concentrations for 24/48 h. (B, D) Cell viability was analyzed by a CCK‐8 assay. The cells were incubated with PTX at different concentrations for 24/48 h. (E, F) Cell proliferation ability was detected by a colony formation assay. The cells were incubated with 1 μmol·L^−1^ PTX or DMDD at different concentrations for 24 h. The assay results were expressed as the relative value of the experimental group/control group. (G, H) Cell migration ability was detected by a transwell assay. The cells were incubated with 1 μmol·L^−1^ PTX or DMDD at different concentrations for 24 h (scale bar = 100 μm). The assay results were expressed as the relative value of the experimental group/control group. DMDD, 2‐dpdecyl‐6‐methoxycyclohexa‐2,5‐diene‐1,4‐dione; PTX, paclitaxel; I, control; II, PTX; III, DMDD‐L; IV, DMDD‐M; V, DMDD‐H. All data are presented as the mean ± SD from three biologically independent experiments, one‐way ANOVA analysis was adopted for multiple comparisons (*P < 0.05, **P < 0.01, ***P < 0.001, vs. Control).

To elucidate the role of DMDD in breast cancer cell proliferation, we assessed cell proliferation in the control and DMDD treatment groups using the MTT assay and the colony formation assay. Cell proliferation experiments demonstrated that DMDD significantly inhibited the proliferation of MDA‐MB‐231 and MCF‐7 cells in a concentration‐ and time‐dependent manner (Fig. 2A–D). The IC_50_ values for MDA‐MB‐231 and MCF‐7 cells treated with DMDD for 24 h were 12.19 μm and 31.17 μm, respectively, whereas the IC_50_ values for 48 h were 11.33 and 26.74 μm, respectively. Additionally, after PTX treatment for 24 h, the average inhibition rate of cell viability was 20%, increasing to 25% after 48 h. The results of the cell cloning experiments indicated that DMDD treatment resulted in a concentration‐dependent decrease in the number of cell colonies (*P* < 0.001) (Fig. 2E,F).

### Effect of DMDD on the migration and invasion of MDA‐MB231 and MCF‐7 cells

**Fig. 3:**
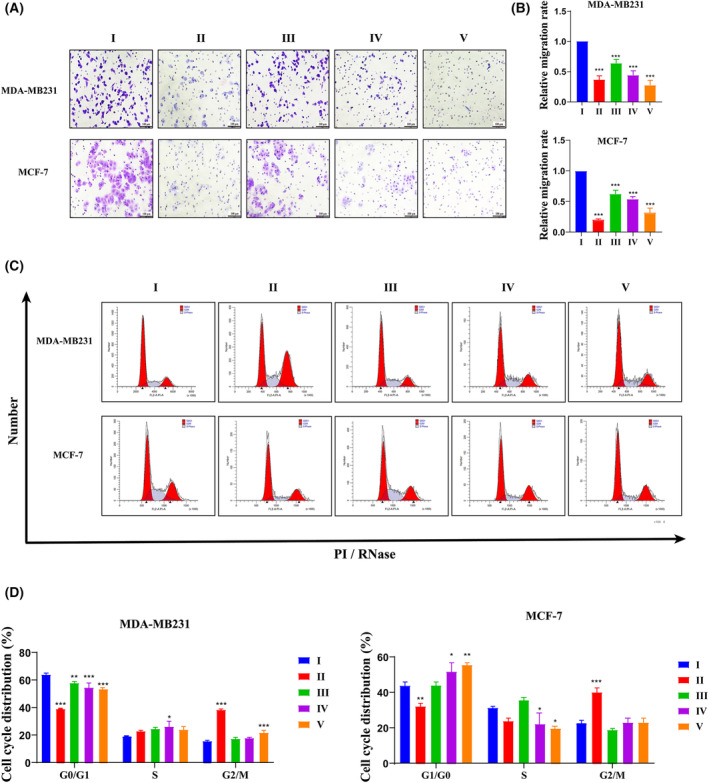
(A, C) Cell invasion ability was detected by transwells assay. The cells were incubated with 1 μmol·L^−1^ PTX or DMDD at different concentrations for 24 h (scale bar = 100 μm). The assay results were expressed as the relative value of the experimental group/control group. (C, D) Cell cycle was detected by flow cytometry. The cells were incubated with 1 μmol·L^−1^ PTX or DMDD at different concentrations for 24 h. DMDD, 2‐dpdecyl‐6‐methoxycyclohexa‐2,5‐diene‐1,4‐dione; PTX, paclitaxel; I, control; II, PTX; III, DMDD‐L; IV, DMDD‐M; V, DMDD‐H. All data are presented as the mean ± SD from three biologically independent experiments, one‐way ANOVA analysis was adopted for multiple comparisons (*P < 0.05, **P < 0.01, ***P < 0.001, vs. Control).

To elucidate the role of DMDD in the migration and invasion of breast cancer cells, we assessed the migratory and invasive abilities of cells in the control and DMDD treatment groups using a transwell assay. After 24 h of DMDD treatment, the migratory ability of the DMDD group was significantly lower than that of the control group (*P* < 0.001) (Fig. 2G,H). Additionally, invasion experiments (Fig. 3A,B) revealed that the number of transmembrane cells in the DMDD group was significantly lower than that in the control group (P < 0.001). Western blotting and qRT‐PCR were employed to assess the expression of PI3K, Akt, mTOR proteins and other factors in each cell line (Fig. 5A–E). The results indicated that DMDD significantly inhibited the expression of these factors compared to the control group. These data suggest that DMDD inhibits the migration and invasion of breast cancer cells.

### Effects of DMDD on cell cycle and apoptosis of MDA‐MB231 and MCF‐7 cells

**Fig. 4:**
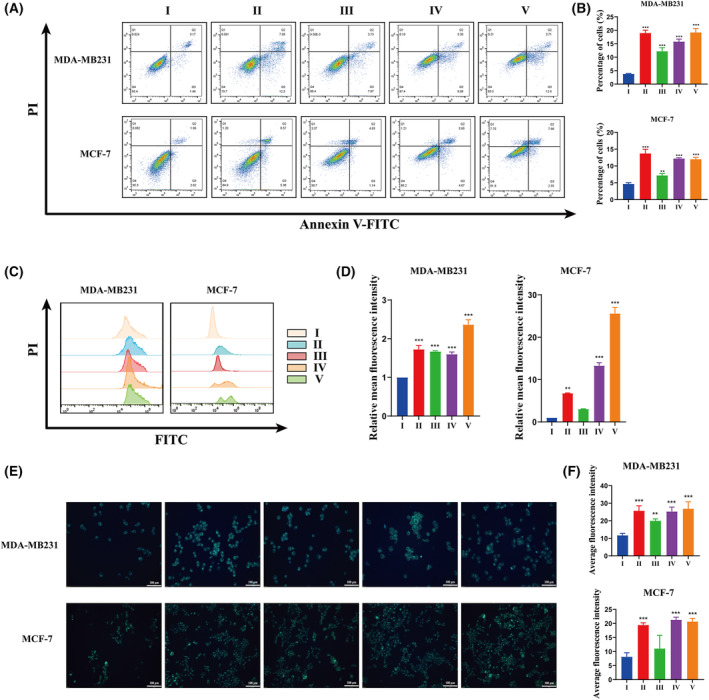
(A, B) Cell apoptosis level was detected by flow cytometry. The cells were incubated with 1 μmol·L^−1^ PTX or DMDD at different concentrations for 24 h. (C, D) ROS levels in cells was detected by flow cytometry. The cells were incubated with 1 μmol·L^−1^ PTX or DMDD at different concentrations for 24 h. The assay results were expressed as the relative value of the experimental group/control group. (E, F) The level of autophagy was detected by a Monodansyla damantanamine‐autophagy detection kit. The cells were incubated with 1 μmol·L^−1^ PTX or DMDD at different concentrations for 24 h. DMDD, 2‐dpdecyl‐6‐methoxycyclohexa‐2,5‐diene‐1,4‐dione; PTX, paclitaxel; I, control; II, PTX; III, DMDD‐L; IV, DMDD‐M; V, DMDD‐H. All data are presented as the mean ± SD from three biologically independent experiments, one‐way ANOVA analysis was adopted for multiple comparisons (*P < 0.05, **P < 0.01, ***P < 0.001, vs. Control).

To reveal the role of DMDD in cell cycle, apoptosis and ROS expression of breast cancer cells, we used flow cytometry to detect cell cycle, apoptosis and ROS levels. The results of the cell cycle experiments indicate that DMDD significantly arrested the cell cycle of MDA‐MB231 cells at the G0/G1 phase and that of MCF‐7 cells at the S phase (Fig. 3C,D). Additionally, the results of the apoptosis assays (Fig. 4A,B) revealed that the apoptosis rates in MDA‐MB231 cells were 10.22% at low concentration, 14.48% at medium concentration and 20.19% at high concentration. In comparison with the control group, the apoptosis rate increased by 3.77%, with a statistically significant difference (*P* < 0.001). In MCF‐7 cells, the apoptosis rates were 7.19% at low concentration, 12.20% at medium concentration and 12.00% at high concentration. Compared with the apoptosis rate of 4.64% in the control group, these differences were statistically significant (*P* < 0.001 for all). Lastly, the results of ROS level detection revealed that, compared to the control group (Fig. 4C,D), the relative fluorescence intensities of ROS in MDA‐MB231 cells were 1.67 at low dose, 1.67 at medium dose and 2.53 at high dose (*P* < 0.001). In MCF‐7 cells, the levels of ROS were significantly higher than those in the control group. The relative fluorescence intensities of ROS were 3.05 at low dose, 13.30 at medium dose and 25.61 at high dose (*P* < 0.001). These data suggest that DMDD induces cell cycle arrest in breast cancer cells, increases ROS levels and promotes apoptosis.

### Effects of DMDD on autophagy in MDA‐MB231 and MCF‐7 cells

Autophagy is a distinct form of programmed cell death, and its inhibition in cancer cells contributes to tumor progression. Therefore, enhancing autophagy in cancer cells inhibits the proliferation and differentiation of tumor cells. The effects of DMDD on MDA‐MB231 and MCF‐7 cells, as well as its impact on autophagy, were assessed using an autophagy vesicle kit (Fig. 4E,F). Mean fluorescence intensity values were utilized to compare changes in autophagy across groups. In MDA‐MB231 cells, the mean fluorescence intensity values for cellular autophagy were 19.98 at low dose, 25.28 at medium dose and 26.88 at high dose (*P* < 0.01, *P* < 0.001 and *P* < 0.001). In MCF‐7 cells, the mean fluorescence intensity values for cellular autophagy were 21.30 at medium dose and 20.69 at high dose (*P* < 0.001). DMDD promotes autophagy in breast cancer cells in a concentration‐dependent manner, leading to cell death.

### Effect of DMDD on PI3K/Akt/mTOR signaling pathway in MDA‐MB231 and MCF‐7 cells

**Fig. 5:**
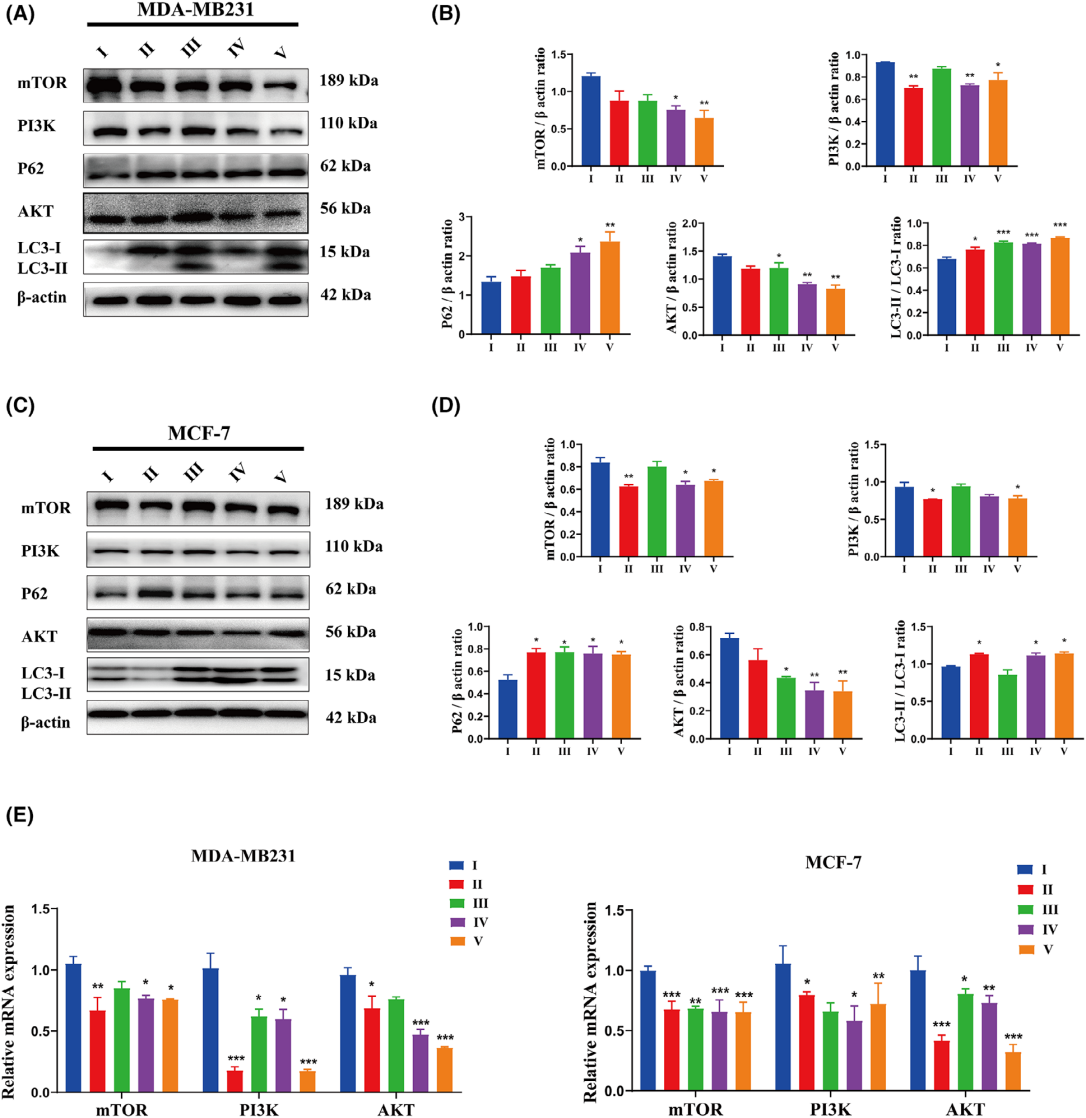
DMDD promoted the proliferation of MCF‐7 and MDA‐MB231 cells through PI3K/Akt/mTOR signaling pathway. (A–D) The protein levels of mTOR, PI3K, Akt, LC3I and LC3II in MCF‐7 and MDA‐MB231 cells were determined using Western blotting. The amount of each product was normalized to the housekeeping gene β‐Actin. The cells were incubated with 1 μmol·L^−1^ PTX or DMDD at different concentrations for 24 h. (E) mTOR, PI3K and Akt mRNA expression in MCF‐7 and MDA‐MB231 cells. The amount of each product was normalized to the housekeeping gene GAPDH. The cells were incubated with 1 μmol·L^−1^ PTX or DMDD at different concentrations for 24 h. DMDD, 2‐dpdecyl‐6‐methoxycyclohexa‐2,5‐diene‐1,4‐dione; PTX, paclitaxel; I, control; II, PTX; III, DMDD‐L; IV, DMDD‐M; V, DMDD‐H. All data are presented as the mean ± SD from three biologically independent experiments, one‐way ANOVA analysis was adopted for multiple comparisons (*P < 0.05, **P < 0.01, ***P < 0.001, vs. Control).

The effects of DMDD on the PI3K/Akt/mTOR signaling pathway in MDA‐MB231 and MCF‐7 cells were analyzed using western blot analysis and qRT‐PCR. The western blot results indicated that, compared to the control group, the protein levels of PI3K, Akt and mTOR in MDA‐MB231 and MCF‐7 cells were down‐regulated, whereas the protein levels of p62 and LC3‐II/LC3‐I were up‐regulated, with statistically significant differences (*P* < 0.05) (Fig. 5A–D). Additionally, the expression levels of PI3K, Akt, and mTOR mRNA were further validated using qRT‐PCR. Furthermore, we found that the expression of PI3K, Akt, and mTOR mRNA in MDA‐MB231 and MCF‐7 cells was significantly decreased after treatment with medium and high doses of DMDD compared to the control group (*P* < 0.05) (Fig. 5E,F).

### DMDD can inhibit the tumor growth of mice with breast cancer

**Fig. 6:**
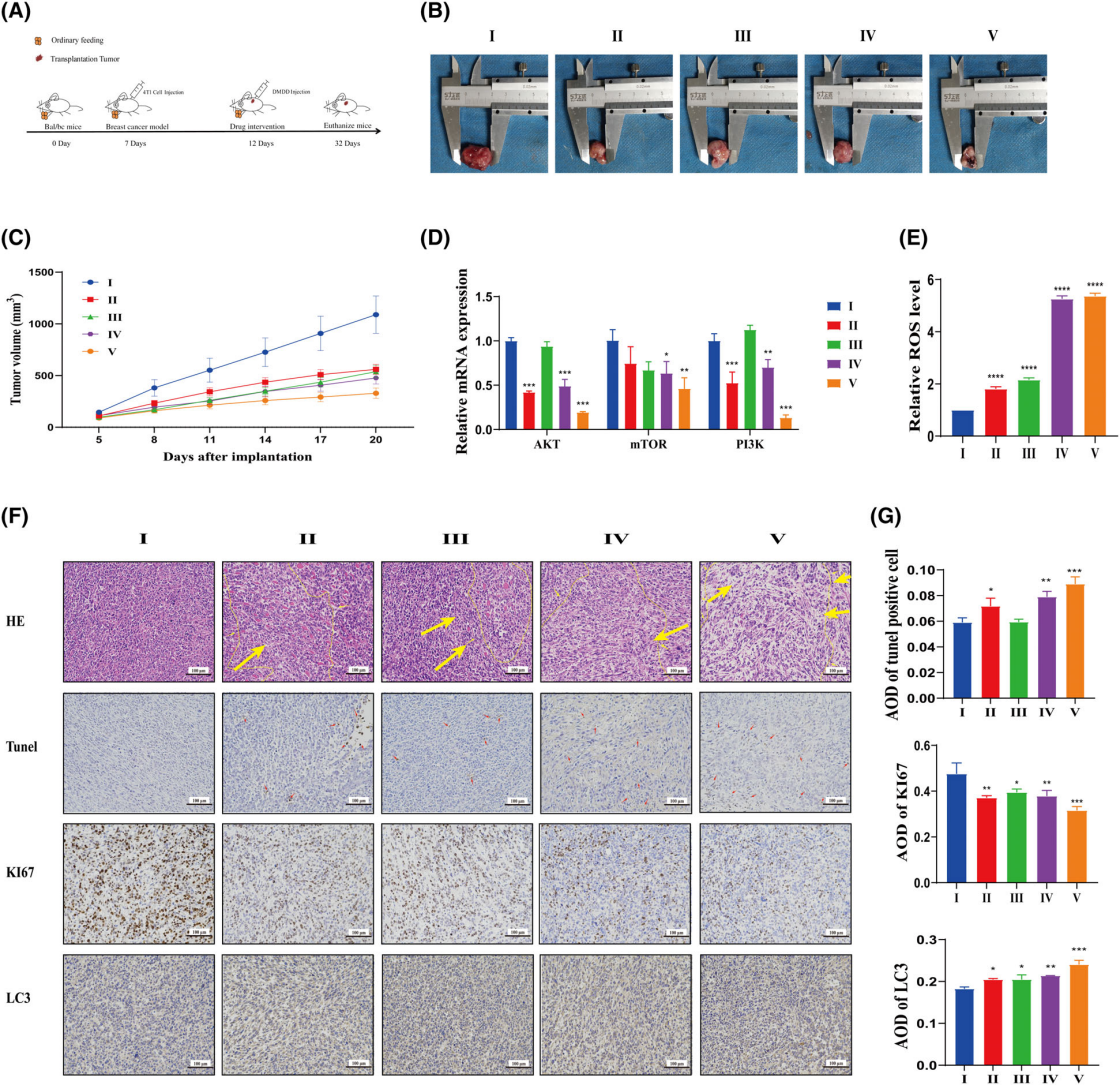
DMDD inhibits tumor growth in breast cancer mice. (A) Construction of mouse breast cancer xenografts and drug intervention timeline. (B) Mouse tumors, from left to right: model group, paclitaxel treatment group, DMDD low, medium and high groups. (C) The tumor growth curve of mouse breast cancer xenografts from day 5 to the day 20; the vertical axis is the tumor volume and the horizontal axis is the growth time. (D) Akt, PI3K and mTOR mRNA expression in mouse tumor tissue. The amount of each product was normalized to the housekeeping gene GAPDH. (E) ROS level in mouse tumor tissue. The assay results were expressed as the relative value of the experimental group/control group. (F, G) HE staining, Tunel staining and immunohistochemistry in mouse tumor tissue; yellow and red arrows refer to the damaged and apoptotic tissues. Scale bar = 100 μm. Paclitaxel, DMDD, 2‐dpdecyl‐6‐methoxycyclohexa‐2,5‐diene‐1,4‐dione. I, model; II, PTX; III, DMDD‐L; IV, DMDD‐M; V, DMDD‐H. All data are presented as the mean ± SD from three biologically independent experiments, one‐way ANOVA analysis was adopted for multiple comparisons (*P < 0.05, **P < 0.01, ***P < 0.001, ****P < 0.0001, vs. model).

The flowchart depicting the establishment of mouse breast cancer graft tumors and the subsequent drug intervention is presented in Fig. 6A. The success rate of the mouse breast cancer xenograft model was 100%. Tumor nodules appeared approximately on day 5, and most tumors reached a volume of 100 mm^3^. Tumor volume increased gradually in the DMDD and PTX groups, not exceeding 1500 mm^3^. Tumors in the model group exhibited a larger size, with a mean volume of 1088.76 mm^3^ (Fig. 6B,C). The tumor inhibition rates in the PTX, DMDD‐L, DMDD‐M and DMDD‐H groups were 69.7%, 48.6%, 50.1% and 56.2%, respectively (Fig. 6C).

### Effect of DMDD on PI3K/Akt/mTOR signaling pathway of mice with breast cancer

The expression levels of PI3K, Akt, and mTOR mRNA in mouse tumor tissues were measured using qRT‐PCR. In the middle and high dose groups of DMDD and PTX, the expression levels of PI3K, Akt and mTOR mRNA were significantly down‐regulated compared to the model group (Fig. 6D).

### Effect of DMDD on ROS of mice with breast cancer

ROS levels in mouse tumor tissues were analyzed using microplate reader. The expression levels of ROS were significantly higher in the DMDD and PTX groups compared to the model group (*P* < 0.0001), showing significant dose‐dependence in the DMDD treatment group (Fig. 6E).

### Effect of DMDD on pathological changes of mice with breast cancer

In this experiment, HE staining, TUNEL staining and immunohistochemical analysis were performed on tumor tissues to investigate the effect of DMDD on apoptosis preliminarily. The HE‐stained sections revealed that tumor cells in the model group were intact, well‐arranged and large in size, exhibiting diverse nuclei and prominent nucleoli, with no inflammatory cell infiltration observed under light microscopy. By contrast, the PTX and DMDD groups exhibited significantly damaged tumor cells that were loosely arranged, with an increased number of apoptotic cells, membrane shrinkage, decreased cell volume, nuclear condensation and chromatin aggregation. The pathological results are shown in Fig. 6F. TUNEL‐stained sections indicated that apoptotic cells were stained brown, whereas the model group exhibited a lack of brown‐stained apoptotic cells. Conversely, the number of brown apoptotic cells increased in the PTX and DMDD groups. Notably, the DMDD‐H group showed a 1.5‐fold increase in the number of apoptotic cells compared to the model group (Fig. 6G). Furthermore, immunohistochemical methods were employed to detect the expression of LC3 and Ki67 in tumor tissues. Positive expression of these proteins was primarily localized in the cytoplasm and parts of the nucleus. Compared to the model group, the expression of Ki67 protein was down‐regulated, whereas LC3 protein expression was elevated in the DMDD and PTX groups, indicating that DMDD may promote autophagic apoptosis and inhibit tumor growth in mouse tumor cells (Fig. 6F,G).

## Discussion

Breast cancer is the most prevalent malignant tumor among women worldwide. Despite significant advancements in the field of breast cancer treatment, the effectiveness of different modalities remains low [23]. An increasing number of anti‐tumor natural active compounds have been discovered due to the growth of natural medicines. Natural active ingredients can prevent tumor growth by targeting various mechanisms, but the underlying mechanism is uncertain. This is an area of active research in the prevention and treatment of cancer and has promising contributions to drug development and application [24]. A compound known as DMDD was identified and extracted from the root of *A. carambola* L. [25]. In recent years, our research team has demonstrated that DMDD can prevent the proliferation of different tumor cell lines, particularly breast cancer cells, *in vitro* [26, 27, 28]. DMDD may therefore be a potent anti‐tumor agent.

Autophagy is an evolutionarily conserved, selective and adaptive response that degrades damaged cellular organelles, pathogens, and other components. It plays an important role in regulating cellular damage, aging, and maintaining homeostasis of the intracellular environment [29]. The recycling process involves transporting excess or damaged cytoplasmic substances to lysosomes for degradation, which occurs through three pathways: microautophagy, chaperone‐mediated autophagy and macroautophagy. Macroautophagy (hereinafter referred to as autophagy) is a complex process that promotes the massive and selective degradation of damaged proteins, organelles, or surplus molecules to produce macromolecular components and provide energy for metabolic pathways [30]. Autophagy involves more than 20 core autophagy proteins, which encapsulate cytoplasmic cargoes in a double‐membrane vesicle structure called autophagosomes [31]. After engulfing the cargo, autophagosomes fuse with lysosomes, and pH‐sensitive hydrolases mediate the degradation of the cargo [30]. Selective autophagy occurs by targeting specific goods (including organelles) to autophagosomes. LC3 and p62 are key autophagy marker proteins. LC3 generally exists in the form of LC3‐I and LC3‐II, with LC3‐II localized in the autophagosome membrane. p62 is the most important cargo protein for selective autophagy, serving as a bridge connecting LC3‐II and the ubiquitylated substrate to be degraded. It fuses with lysosomes to form autophagic lysosomes, which are cleared after entering the autophagosome [32, 33]. LC3 expression is positively correlated with the level of autophagy, whereas the expression of the autophagy adapter p62, a ubiquitination protein scaffold, is negatively correlated with autophagy levels. p62 can mediate the degradation of ubiquitinated substrates by autophagosomes through its binding to LC3‐II. Autophagy is involved in the pathogenesis of many diseases, and abnormalities in autophagy can contribute to the development of cancer [34]. Autophagy can both promote and inhibit tumor growth under different conditions [35, 36]. For example, gene inhibition of Atg5 or Atg7 has demonstrated that the absence of autophagy in tumor‐prone mouse models increases the incidence of precancerous lesions and tumors after activation of the RAS pathway, indicating that autophagy can prevent cancer development [37, 38, 39, 40]. As our study found, DMDD significantly upregulated the number of autophagic vesicles and the expression of LC3‐II and p62 proteins at the same time as downregulating the expression of Akt and mTOR. This suggests that DMDD‐induced autophagy may be closely related to the inhibition of the PI3K/Akt/mTOR signaling pathway. Furthermore, the transient increase in p62 levels may result from the short‐term aggregation of p62 that has not yet been degraded by autophagosomes as a result of enhanced upstream expression of autophagy. Zhao *et al*. [32] also found that LC3‐II and p62 protein expression increased in their study of silica nanoparticle‐induced A549 cell injury. Other studies have shown that ROS‐mediated enhancement of death receptors promotes autophagy via NF‐κB‐mediated p62 overexpression [41], which may also contribute to elevated p62 protein levels.

The PI3K/Akt/mTOR pathway plays a crucial role in regulating cell proliferation, migration, apoptosis, and autophagy [42]. Abnormal activation of this pathway has been observed in various types of cancer. The PI3K catalytic subunit p110α is frequently mutated or amplified in common human cancers, including breast cancer, colon cancer and lung cancer. Another key component of this pathway is Akt, a serine/threonine kinase that belongs to the AGC kinase family. In particular, Akt‐1 is associated with a common mutation in breast cancer known as E17K (AKT1 mutations). After PI3K activation, Akt is phosphorylated, which subsequently activates the downstream signaling molecule mTOR. The mTOR complex is mainly divided into mTORC1 and mTORC2. mTORC1 is sensitive to nutrients and is involved in the regulation of cellular lipid metabolism, glycolysis and autophagy, whereas mTORC2 is related to cytoskeleton remodeling, cell activation, and regulation of movement and angiogenesis [43]. However, mTOR is often overexpressed in tumor cells, leading to vigorous glycolytic metabolism that promotes tumor growth, metastasis and invasion of healthy tissues. Inhibition of mTOR function can deactivate the PI3K/Akt/mTOR pathway and induce autophagy in tumor cells [44, 45, 46]. Furthermore, it has been found that the excessive accumulation of ROS in tumor cells with abnormal metabolism has a significant anti‐cancer effect, placing the cells in a state of oxidative stress. ROS can directly regulate the phosphorylation of Akt, which in turn controls the entire PI3K/Akt signaling pathway, an important component of the pro‐apoptotic activity of various anti‐tumor drugs [47]. Increased intracellular ROS levels can inhibit the tumor cell cycle in the G1 phase and trigger apoptosis [48, 49]. Moreover, tumor necrosis factor‐related apoptosis receptors, as well as ROS‐mediated death receptors, are enhanced. Malignant tumors such as liver cancer, bladder cancer and gastric cancer are affected by these mechanisms [50, 51]. This phenomenon was also observed in the present study, where DMDD effectively inhibited the expression of PI3K, Akt and mTOR in MDA‐MB‐231 and MCF‐7 cells. Additionally, DMDD increased ROS levels in breast cancer cells and mouse tumor tissues at the same time as inhibiting the proliferation of breast cancer cells and mouse tumors. These results highlight the potential of DMDD as a therapeutic agent for breast cancer by targeting the PI3K/Akt/mTOR pathway and inducing autophagy in tumor cells.

In summary, the present study demonstrates that DMDD inhibits the PI3K/Akt/mTOR signaling pathway by downregulating the expression of PI3K, Akt and mTOR. It promotes the expression of ROS in breast cancer MDA‐MB‐231 and MCF‐7 cells at the same time as inhibiting migration, invasion, metastasis and autophagy. These results suggest that DMDD may be a potential therapeutic agent, with the downregulation of PI3K, Akt and mTOR representing a viable strategy for treating ER‐α positive MCF‐7 and triple‐negative MDA‐MB‐231 breast cancers. Given the significant correlation between DMDD and breast cancer, our future research may focus on other breast cancer subtypes, such as luminal A and luminal B, aiming to verify the pharmacological effects and efficacy of DMDD on these related subtypes.

## Conflicts of interest

The authors declare that they have no conflicts of interest.

### Peer review

The peer review history for this article is available at https://www.webofscience.com/api/gateway/wos/peer‐review/10.1002/2211‐5463.13940.

## Author contributions

LC contributed to writing the original draft. LC, RH, GH, CH and TTHP contributed to methodology. LC, MC, YX, YZ, SM, YH, TL and YL contributed to investigations. LC, MC, CH and TTHP contributed to the formal analysis. LC, MC and CH contributed to data curation. MC, CH and TTHP contributed to reviewing and editing. YX and YZ contributed to validation. RH, GH, CH and TTHP contributed to supervision. TTHP and GH contributed to resources, project administration, funding acquisition and conceptualization.

## Data Availability

The data that support the findings of this study are available from the corresponding author upon reasonable request.
